# The New Poor-Law Orders

**Published:** 1914-01-10

**Authors:** 


### THE NEW POOR-LAW ORDERS.

It is unnecessary to emphasise here the extreme importance of the two new Poor-Law Orders, of which we publish an expert summary elsewhere in this issue. Readers who turn to that summary will also see that we reserve our final criticism until a later issue, when the full effect of the new Orders is realised by the institutions generally, and debated points have been raised, as we expect, in our own correspondence columns. But while judgment may be reserved as to matters of detail, one or two broad points demand immediate attention.

In the first place it is fair to say that the new Orders form a remarkable tribute to the cogency of the criticisms which were levelled in The Hospital at the time when the Draft Order made its appearance. The new Orders are not, it will be seen, to apply to separated infirmaries or to separate institutions for children, and that may be taken as showing that it is in unseparated infirmaries, as those acquainted with the facts well know, that difficulties persist. No better argument for complete separation could be found. Our criticism of the Draft Order fastened on the loose phraseology employed, and we are glad to note that the new Orders are much better drafted in this respect. It is further to be noted that the duties of the medical officers are to be increased by the keeping of record papers, on the lines already customary in the voluntary hospitals. The increase of work here indicated should prove also an improvement, when it is remarked that the workhouse medical relief book is to be abolished. For the points involved in this administrative change we refer our readers to the summary already mentioned, while we turn now to the success of our criticisms in another direction.

Under the Draft Order the rights of inmates in the institutions were none too well guarded. Too much was left in the discretion of the workhouse master, and too little to the direct responsibility of the medical officer. On this point the criticisms of the institutional press have proved of some effect, and it is now to be noted that the employment of inmates in many instances has been forbidden except in ways and under conditions which the medical officer may lay down. There is no doubt that successful administration and the position and well-being of the inmates will gain under the provisions which place them more intimately than before under the medical officer's care. The same holds good of the punishment of inmates who transgress discipline in this or that respect. Here the control of the medical officer is again increased, and the increase should prove welcome.

The Nursing Order it is not our peculiar purpose to examine in great detail, but readers of the summary referred to will note here again a substantial victory for our criticisms. The superintendent nurse's responsibility is increased; high qualifications, including a midwifery training, are insisted on; and the era of her freedom from lay control in her own skilled department has begun officially to dawn. The moral of the new Orders then is that institutional workers in Poor-Law infirmaries have only to combine through their own organs to secure the attention of the Local Government Board, and, in time, the granting of those basic reforms on which the most experienced of them are set. That should be sufficient encouragement for the Poor-Law world to begin the New Year with, and we hope that it may prove a spur to greater endeavour. The new Orders will not prove the last word that the Local Government Board has to say on infirmary administration. What the last word will be it rests with the institutional officers through repeated argument in our columns, and wherever else opportunity occurs, to decide. Where criticism has won the modifications here patent, it has only to repeat its efforts to complete the administrative reforms which the well-being of the modern infirmary requires.

